# Stevia Leaf Extract Fermented with Plant-Derived *Lactobacillus plantarum* SN13T Displays Anticancer Activity to Pancreatic Cancer PANC-1 Cell Line

**DOI:** 10.3390/ijms26094186

**Published:** 2025-04-28

**Authors:** Rentao Zhang, Narandalai Danshiitsoodol, Masafumi Noda, Sayaka Yonezawa, Keishi Kanno, Masanori Sugiyama

**Affiliations:** 1 Department of Probiotic Science for Preventive Medicine, Graduate School of Biomedical and Health Sciences, Hiroshima University, Hiroshima 734-8551, Japan; d225530@hiroshima-u.ac.jp (R.Z.); naraa@hiroshima-u.ac.jp (N.D.); bel@hiroshima-u.ac.jp (M.N.); 2 Department of General Internal Medicine, Hiroshima University Hospital, Hiroshima University, Hiroshima 734-8551, Japan; sayaka22@hiroshima-u.ac.jp (S.Y.); k_keishi@yahoo.com (K.K.)

**Keywords:** apoptosis, chlorogenic acid methyl ester, cytotoxic effect, *Lactobacillus plantarum*, PANC-1 cell line, stevia

## Abstract

Pancreatic cancer is a highly malignant tumor that remains a significant global health burden. In this study, we demonstrated the anticancer potential of stevia leaf extract fermented with plant-derived *Lactobacillus* (*L*.) *plantarum* SN13T strain. Evaluation of antioxidant capacity (including DPPH and ABTS radical scavenging activities and H_2_O_2_-induced oxidative damage repair in HEK-293 cells), as well as cytotoxicity against pancreatic cancer cells (PANC-1) and non-cancerous human embryonic kidney (HEK-293), revealed that the fermented extract exhibited significantly enhanced antioxidant activity and cytotoxicity against PANC-1 cells while showing minimal toxicity to HEK-293 cells compared to the unfermented extract. Further, validation through clonogenic, migration, and wound-healing assays demonstrated that the fermented extract effectively inhibited the proliferation and migration of PANC-1 cells. The active compound in the fermented extract has been identified as chlorogenic acid methyl ester (CAME), with a concentration of 374.4 μg/mL. Flow cytometry analysis indicated that CAME significantly arrested PANC-1 cells in the G_0_/G_1_ phase and induced apoptosis. Furthermore, CAME upregulated the expression of pro-apoptotic genes *Bax*, *Bad*, *Caspase-3*/*9*, *Cytochrome c*, and *E-cadherin*, while downregulating the anti-apoptotic gene *Bcl-2*. These findings suggest that CAME exerts potent cytotoxic effects on PANC-1 cells by inhibiting cell proliferation and migration, arresting the cell cycle, and regulating apoptosis-related gene expression. In conclusion, stevia leaf extract fermented with *L. plantarum* SN13T, which contains CAME, may serve as a promising candidate for pancreatic cancer treatment.

## 1. Introduction

Pancreatic cancer is a highly malignant tumor of the digestive system with a poor prognosis. Globally, the incidence and mortality rates of pancreatic cancer continue to rise, with a five-year survival rate of less than 10% [1]. The primary reason pancreatic cancer is considered one of the deadliest cancers is its subtle, insidious onset, with most patients being diagnosed at an advanced stage and missing the optimal treatment window [2,3,4]. Furthermore, pancreatic cancer is highly invasive and prone to metastasis, showing significant resistance to existing treatments such as surgery, radiotherapy, and chemotherapy, resulting in very limited therapeutic efficacy [2,3,5,6,7]. Therefore, there is an urgent need to identify new and effective anticancer compounds, particularly those derived from medicinal plants.

Stevia (*Stevia rebaudiana* Bertoni) is a perennial herb widely utilized in the food and pharmaceutical industries, valued not only for its intense natural sweetness but also for its potential health-promoting properties. In addition to steviol glycosides, stevia leaves are rich in flavonoids and phenolic compounds, which exhibit various biological activities, including antioxidant, anti-inflammatory, antihypertensive, and anticancer effects [8,9,10,11]. While purified steviol glycosides and their derivatives (e.g., stevioside, isosteviol) have demonstrated cytotoxic and antiproliferative activities against several cancer cell lines [12,13], extracts from the stevia leaf also exhibit similar, though less potent, anticancer effects. For example, Vaško et al. reported that an aqueous extract of stevia only reduced the viability of Caco-2 and MCF-7 cells at high concentrations (1000 μg/mL), indicating the limited efficacy of the crude extract [14]. Similarly, Lopez et al. showed that ethanol extracts of stevia leaves exhibited dose-dependent antiproliferative and antioxidant effects in HeLa cells, with the former showing higher cytotoxicity [11]. However, despite the promising anticancer potential demonstrated by stevia leaf extract, effectively utilizing its bioactive components remains challenging.

To enhance the pharmacological efficacy of natural plant extracts, microbial biotransformation has emerged as a promising strategy. This process utilizes microbial enzymes and coenzymes to structurally modify plant-derived compounds—via hydrolysis, acylation, dehydrogenation, and other reactions—generating secondary metabolites with improved bioactivity [15]. Among microbial agents, lactic acid bacteria (LAB) are widely applied due to their Generally Recognized As Safe (GRAS) status, ability to produce bioactive metabolites, and capacity to modulate gut microbiota and maintain microecological balance [16,17]. Previous studies have shown that LAB fermentation can enhance the anticancer potential of various plant materials. For example, *Lactobacillus* (*L.*) *plantarum* YT013 exerts a strong and sustained cytotoxic effect on gastric cancer cells by synthesizing exopolysaccharides [18]. Barley extract fermented with *L. plantarum* dy-1 showed a stronger inhibitory effect on colon cancer HT-29 cells compared to its non-fermented form [19]. Similarly, the fermentation of *Panax notoginseng* with LAB increased its antiproliferative activity against liver cancer cells by enriching bioactive ginsenosides such as Rg3 and Rh1 [20].

Our research team has isolated over 1200 plant-derived LAB strains from fruits, vegetables, flowers, and medicinal plants and evaluated their health benefits. In this study, we investigated the ability of *L. plantarum* SN13T, a strain isolated from banana leaves, to enhance the antioxidant and anticancer activities of stevia leaf extract through fermentation. By comparing fermented and non-fermented extracts, we aimed to identify the key compounds responsible for any observed enhancement in bioactivity and provide a new approach to improving the efficacy of herbal medicine in cancer prevention and therapy.

## 2. Results and Discussion

### 2.1. Optimization Results of the Fermentation Process for Stevia Leaf Extract

The growing demand for pharmacologically active natural products has driven the development of microbial transformation techniques, thereby promoting the efficient production of natural products and the discovery of novel bioactive substances [21]. However, microbial transformation is influenced by various factors, including the fermentation method, culture medium, temperature, humidity, duration, pH, agitation speed, substrate solubility, and the presence of cofactors [21,22,23]. Therefore, improving and optimizing these techniques has become a key strategy to enhance both the yield and bioactivity of the transformed products. Such optimization not only increases the production efficiency of target compounds but also promotes the discovery of new bioactive molecules, thereby expanding the pharmacological potential of natural products.

**Figure 1:**
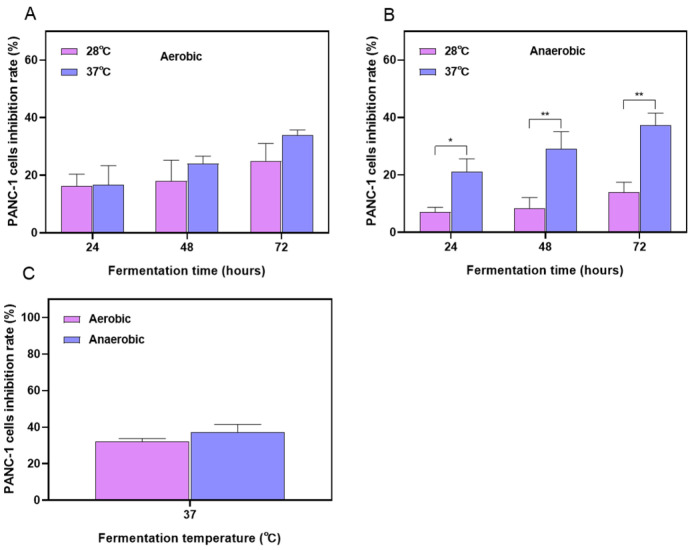
The effects of fermented extract obtained under different culture conditions on the activity of PANC-1 cells. The impact of FSLE (250 μg/mL) on PANC-1 cells’ viability when cultured at 28 °C or 37 °C for 24, 48, and 72 h under aerobic (**A**) or anaerobic (**B**) conditions. (**C**) The effect of FSLE (250 μg/mL) on PANC-1 cells’ activity after 72 h of culture at 37 °C under aerobic and anaerobic conditions. * *p* < 0.05, ** *p* < 0.01, indicated a significant difference.

To identify the optimal growth conditions for maximizing product yield and/or improving product quality, we optimized the fermentation time, temperature, and aeration conditions. The effectiveness of these optimizations was evaluated by measuring the impact of fermentation products on the activity against pancreatic cancer cells. Figure 1A and B, respectively, show that under either aerobic or anaerobic conditions, anti-PANC-1 cell activity of the fermentation products increased with longer fermentation times. At the same fermentation time, fermentation at 37 °C resulted in greater cytotoxic activity than at 28 °C, indicating that 37 °C is more conducive to the production of bioactive compounds. Studies have shown that enzyme activity is typically reduced at lower temperatures, while an increase in temperature enhances catalytic enzyme activity, leading to higher catalytic efficiency [24]. Previous research has also demonstrated that, under adequate nutrient and growth factor conditions, the accumulation of metabolic products increased with extended incubation time [25]. As shown in Figure 1C, the impact of aerobic versus anaerobic cultivation on the activity of fermentation products was compared under the condition of 72 h fermentation. We observed that, at the fermentation temperature of 37 °C, the anaerobic fermentation extract exhibited higher cytotoxicity against PANC-1 cells; however, the difference between the two conditions was not statistically significant. Eventually, we determined that anaerobic fermentation at 37 °C for 72 h was the optimal condition.

### 2.2. Cytotoxicity Evaluation In Vitro

**Figure 2:**
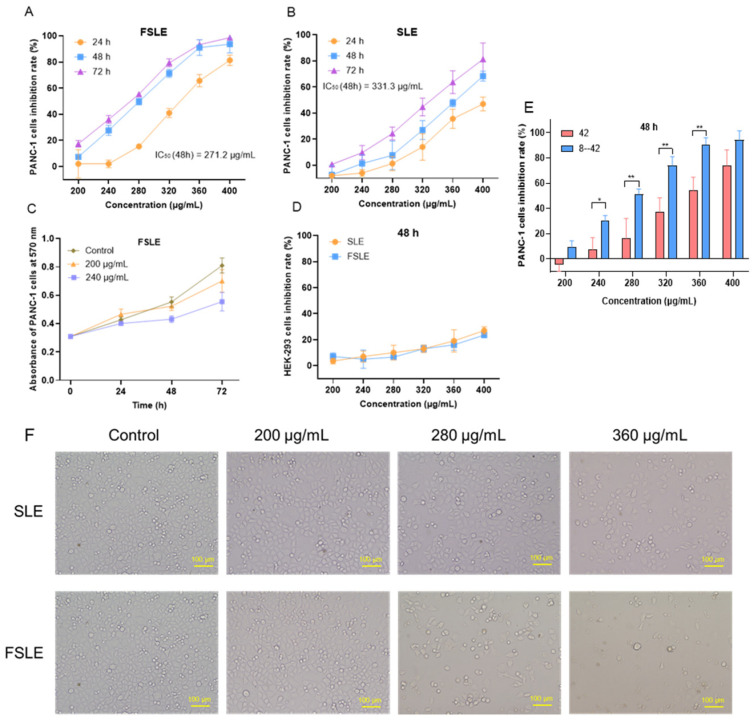
Toxic effects of SLE and FSLE on PANC-1 and HEK-293 cells. (**A**) Cytotoxicity of FSLE on PANC-1 cells after 24, 48, and 72 h. (**B**) Cytotoxicity of SLE on PANC-1 cells after 24, 48, and 72 h. (**C**) Absorbance of PANC-1 cells treated with FSLE at 200 and 240 μg/mL after 24, 48, and 72 h. (**D**) Cytotoxic effects of SLE and FSLE on HEK-293 cells after 48 h. (**E**) Cytotoxicity of FSLE or SLE on PANC-1 cells after 48 h. (**F**) Morphological changes in PANC-1 cells treated with SLE and FSLE at 200, 280, and 360 μg/mL after 48 h. * *p* < 0.05, ** *p* < 0.01, compared SLE with FSLE. Scale bar = 100 μm.

To evaluate the cytotoxic effects of fermented stevia leaf extract (FSLE) and stevia leaf extract (SLE), we tested their activity on pancreatic cancer PANC-1 cells and non-cancerous human embryonic kidney HEK-293 cells. As shown in Figure 2A,B, both SLE (IC_50_ (48 h) = 331.3 μg/mL) and FSLE (IC_50_ (48 h) = 271.2 μg/mL) significantly reduced the viability of PANC-1 cells in a concentration- and time-dependent manner. Notably, FSLE exhibited stronger cytotoxicity than SLE at equivalent concentrations (Figure 2E), suggesting that fermentation enhanced the bioactivity of the extract. Comparative observations of cell morphology revealed that both FSLE and SLE treatments resulted in reduced cell number, weakened cell adhesion, and morphological changes such as cell rounding and shrinkage. However, FSLE exerted a more pronounced effect than SLE, as evidenced by a greater degree of cell detachment and dispersion at the same treatment concentrations and time points (Figure 2F). Numerous studies have shown that fermentation with lactic acid bacteria enhances the biological activity of herbal products by increasing the content of more active metabolites. For instance, Jiang et al. reported that fermented ginseng exhibited stronger immune-enhancing and anticancer properties than its non-fermented counterpart [26]. Similarly, Le et al. found that lactic acid bacteria–fermented soy milk significantly increased the levels of genistein and daidzein, thereby enhancing its inhibitory effect on human colon cancer Caco-2 and HCT116 cells [27]. Barley extract fermented with *L. plantarum* de-1 also exhibited stronger growth-inhibitory activity against human colon cancer HT-29 cells [19].

Interestingly, at concentrations of 200 and 240 μg/mL, the number of viable PANC-1 cells still increased over time (Figure 2C), suggesting that under these conditions, FSLE primarily inhibited cell proliferation rather than directly inducing cell death. Importantly, FSLE demonstrated lower toxicity toward HEK-293 cells, with minimal inhibition observed even at the highest concentration tested (Figure 2D). This selective cytotoxicity implies a favorable safety profile, consistent with previous reports. For example, Lizardo et al. found that lactic acid bacteria–fermented cherry elderberry extract selectively inhibited SW480 colon cancer cells without affecting normal human epithelial HaCat cells [28]. Taken together, these findings indicate that fermentation with *L. plantarum* SN13T significantly enhances the antitumor activity of the stevia leaf extract.

### 2.3. In Vitro Determination of Antioxidant Capacity

**Figure 3:**
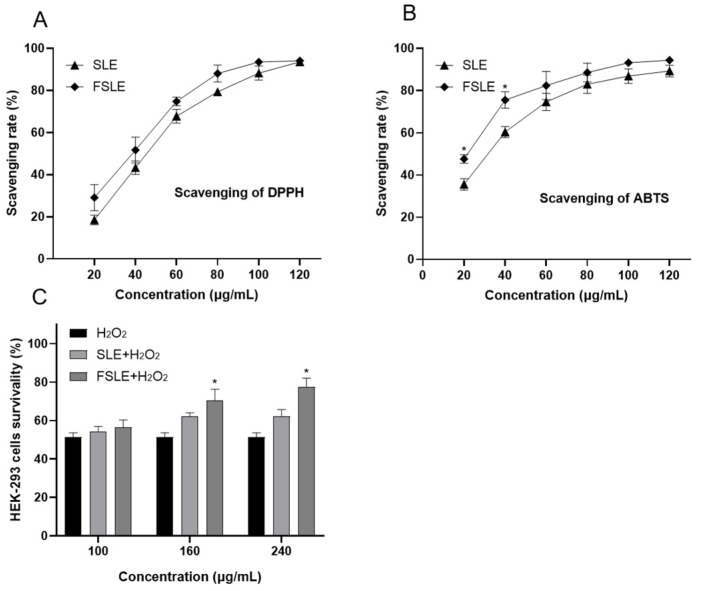
Antioxidant activity of SLE and FSLE. In vitro radical scavenging activity of SLE and FSLE: DPPH radical (**A**) and ABTS radical (**B**). (**C**) Effect of SLE and FSLE on HEK293 cell viability after H_2_O_2_-induced oxidative stress following pretreatment. * *p* < 0.05, compared SLE with FSLE.

To further evaluate the antioxidant capacity of FSLE in vitro, we measured its ability to scavenge DPPH and ABTS radicals and examined its protective effect against H_2_O_2_-induced oxidative stress in cells. The DPPH radical is a stable free radical commonly used to assess the radical scavenging ability of antioxidants [29]. As shown in Figure 3A, FSLE exhibited a dose-dependent increase in DPPH radical–scavenging activity. At a concentration of 120 μg/mL, the scavenging rate reached 94.05%. Within the tested concentration range (20–120 μg/mL), FSLE demonstrated stronger scavenging activity than SLE, indicating its enhanced ability to donate electrons or terminate radical chain reactions. Similarly, the ABTS radical–scavenging assay, another widely accepted method for measuring antioxidant activity [29], showed that FSLE had superior performance compared to SLE. As shown in Figure 3B, FSLE displayed a concentration-dependent increase in ABTS radical–scavenging activity. At 40 μg/mL, FSLE achieved a scavenging rate of 75.5%, significantly higher than the 60.3% observed for SLE.

To further assess the biological relevance of this antioxidant activity, we examined the protective effects of FSLE against H_2_O_2_-induced oxidative stress in HEK293 cells. As shown in Figure 3C, exposure to 500 μM H_2_O_2_ significantly reduced cell viability to 51.5%. Treatment with FSLE provided a concentration-dependent protective effect, increasing cell viability to 56.5, 70.5, and 77.8% at low, medium, and high concentrations, respectively. In contrast, SLE treatment under the same conditions restored cell viability to 54.2, 62.3, and 63.5%, indicating a relatively weaker protective effect.

Previous studies have reported comparable antioxidant effects of stevia leaf extract. López et al. demonstrated that pretreatment with 15.62 μg/mL of stevia ethanol extract increased the antioxidant capacity of HepG2 cells by 10.9% [11]. Latha et al. found that 200 μg/mL stevia aqueous ethanol extract achieved a 67.67% scavenging rate of H_2_O_2_ and alleviated LPS-induced oxidative liver damage in rats at a dose of 250 mg/kg [30]. Additionally, polysaccharides extracted from stevia roots exhibited similar effects by protecting RAW264.7 cells from H_2_O_2_-induced damage through inhibition of lipid peroxidation and enhancement of antioxidant enzyme activity [31].

Both SLE and FSLE exhibited notable antioxidant and cytoprotective effects; however, FSLE consistently outperformed SLE in all tested assays. These antioxidant properties are largely attributed to polyphenolic compounds such as chlorogenic acid (CA) and various flavonoids, which are well known for their free radical–scavenging capacities [9,11]. The superior efficacy of FSLE is likely due to the biotransformation of native phenolic compounds during fermentation. In particular, the conversion of CA into its methylated derivative CAME and the generation of other unidentified metabolites with stronger radical-scavenging potential may contribute to the increased antioxidant capacity of FSLE. These findings align with our previous study, where fermentation of mint extract with *L. plantarum* SN13T significantly enhanced its antioxidant and anti-inflammatory activities compared to the non-fermented extract in LPS-induced RAW264.7 macrophages [23].

### 2.4. Effects of FSLE on the Proliferation and Migration of PANC-1 Cells

**Figure 4:**
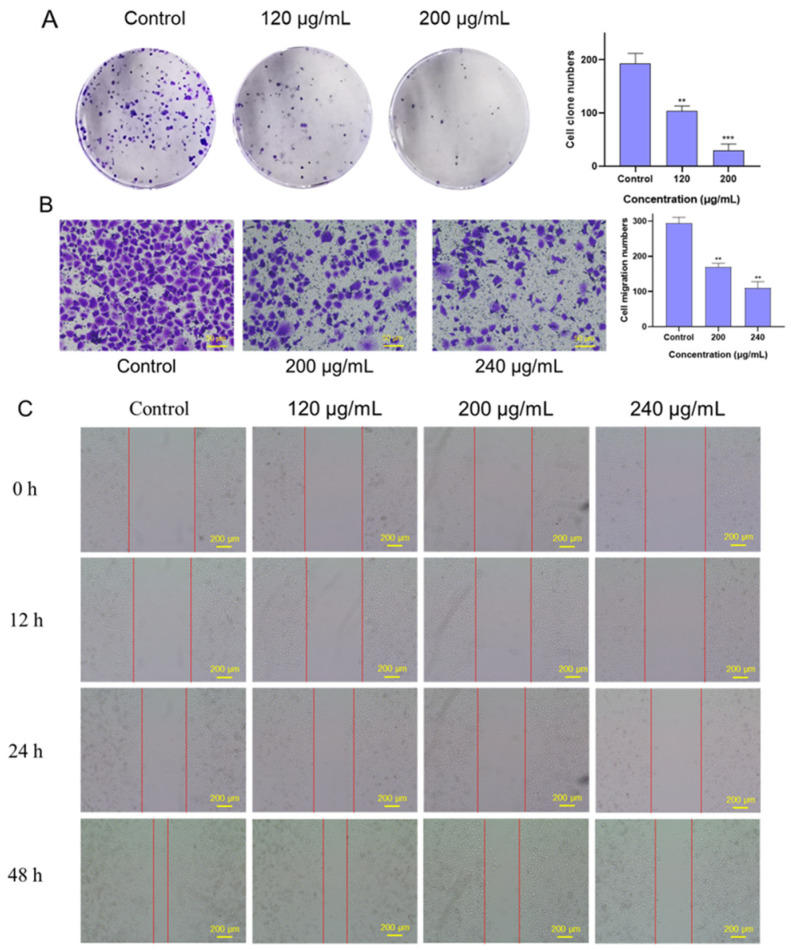
Effects of FSLE on the proliferation and migration of PANC-1 cells. (**A**) FSLE inhibits the proliferation of PANC-1 cells after 10 d of treatment. (**B**) The impact of FSLE on PANC-1 cells’ migration after 48 h of treatment. (**C**) Changes in PANC-1 cells’ migration with different concentrations of FSLE over varying treatment durations. ** *p* < 0.01, *** *p* < 0.001 as compared with control.

We evaluated the effect of FSLE on the proliferation of PANC-1 cells using a colony formation assay. As shown in Figure 4A, FSLE treatment significantly reduced colony numbers in a dose-dependent manner, with higher concentrations resulting in fewer colonies. Since cell migration and invasion play critical roles in the process of tumor metastasis [32], we investigated the effect of FSLE on the migratory ability of PANC-1 cells using a transwell migration assay. The results showed that FSLE significantly inhibited PANC-1 cell migration in a concentration-dependent manner (Figure 4B). After 24 h of treatment, the number of cells that migrated to the bottom of the chamber was significantly reduced, was markedly reduced, and this reduction was positively correlated with the concentration of FSLE. Moreover, CAME significantly upregulated the gene expression of *E-cadherin* at the molecular level, further supporting the inhibitory effect of FSLE on PANC-1 cells’ migration. The role of FSLE in inhibiting both the proliferation and migration of PANC-1 cells was further confirmed through a wound-healing assay. The results in Figure 4C further confirmed that FSLE significantly inhibited the migration ability of PANC-1 cells. As the incubation time with FSLE increased from 0 to 48 h, the reduction in the width of the cell-free gap was markedly less pronounced in FSLE-treated groups compared to the control group, where the gap was almost completely closed. In summary, FSLE significantly inhibited both the proliferation and migration of PANC-1 cells.

### 2.5. Analysis of Antitumor Active Components in FSLE

**Figure 5:**
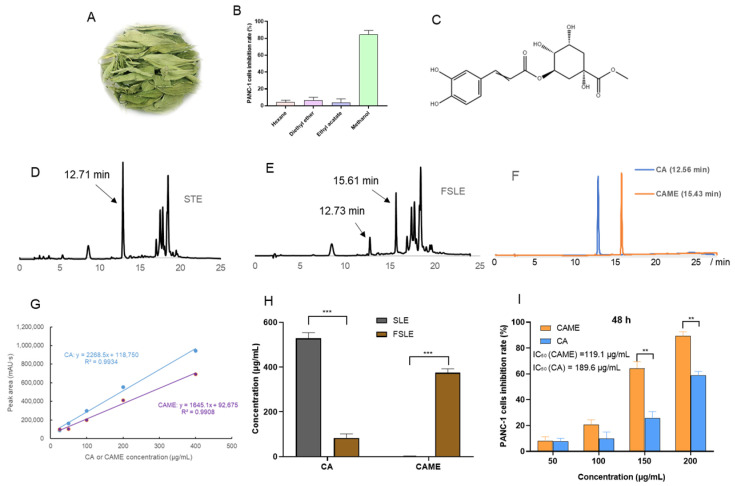
Comparison of HPLC chromatograms of stevia leaf extract before and after fermentation, changes in the active compound content, and its cytotoxicity against PANC-1 cells. (**A**) Dried leaves of stevia. (**B**) Cytotoxicity of different extracted fractions on PANC-1 cells. (**C**) The chemical structure of CAME. (**D**) HPLC chromatogram of SLE. (**E**) HPLC chromatogram of FSLE. (**F**) HPLC chromatogram of CA and CAME. (**G**) Standard curves of CA and CAME. (**H**) The change in the content of CA and CAME in the stevia leaf extract before and after fermentation. (**I**) CAME and CA cytotoxicity on PANC-1 cells at 48 h. ** *p* < 0.01, *** *p* < 0.001, indicated a significant difference.

Since FSLE exhibited stronger anti-pancreatic cancer PANC-1 cell activity as compared to SLE, we proceeded to isolate its active compounds. FSLE was extracted sequentially with chloroform and methanol. The methanol fraction was then subjected to silica gel column chromatography to separate fractions potentially responsible for the observed cytotoxicity. Cytotoxicity assays in PANC-1 cells were performed to screen the bioactive fractions (Figure 5B). The results showed that the active compounds were extracted and enriched in a methanol solvent.

The most active fraction was further purified by HPLC, leading to the isolation of the major active compound. We compared the HPLC chromatograms of FSLE and SLE, as shown in Figure 5D,E. A new peak at a retention time of 15.61 min was observed in FSLE, which was absent in the SLE profile. Meanwhile, the peak at 12.71 min in SLE was markedly diminished in FSLE. The accumulation of this metabolite in FSLE could be responsible for higher bioactivity. For the identification, this purified compound was analyzed for ESI–MS, ^1^H-NMR, and ^13^C-NMR spectra, as shown in Figure S1a, S1b, and S1c, respectively. A molecular ion was observed at *m*/*z* 369.11 in the positive ion ESI–MS spectrum of the underivatized compound, corresponding to the molecular formula C_17_H_21_O_9_. In the ^1^H-NMR spectrum, δH values were observed at 7.49, 6.77, 6.18, 5.25, and 3.68 ppm, while in the ^13^C-NMR spectrum, δC signals appeared at 175.44, 168.26, 149.66, 147.18, 146.84, 127.63, 122.96, 116.52, 72.56, 38.41, and 38.02 ppm. Taken together, the NMR and MS data were consistent with previously reported values for chlorogenic acid methyl ester [33], whose chemical structure is shown in Figure 5C. As shown in Figure 5F, the retention times of the CA and CAME standards were 12.60 ± 0.1 min and 15.50 ± 0.1 min, respectively. The peak at 12.71 min was consistent with the retention time of CA, while the peak at 15.61 min matched that of CAME. The identification was confirmed based on the consistency of retention times with those of the authentic standards. Quantification of CA and CAME before and after fermentation was performed using external standard curves constructed from serial dilutions of CA and CAME standards (25–400 μg/mL). The resulting equations were as follows (Figure 5G): CA (y = 2268.5x + 118,750, R^2^ = 0.9934); CAME (y = 1645.1x + 92,675, R^2^ = 0.9908), where y represents the peak area and x the concentration (μg/mL). These standard curves were subsequently used to determine the concentrations of CA and CAME in the samples.

As shown in Figure 5H, the concentration of CAME produced after fermentation was 374.4 μg/mL. Based on the fermentation volume of 10 mL, the total yield of purified CAME obtained through column chromatography was 2.88 mg. Interestingly, a significant decrease in CA concentration was observed after fermentation, dropping from 528.3 μg/mL to 86.1 μg/mL. These findings strongly suggest that CA underwent microbial biotransformation during fermentation, most likely catalyzed by methyltransferase enzymes secreted by *L. plantarum* SN13T [34]. This enzymatic activity resulted in the formation of CAME, which was not present in SLE. The transformation of CA to CAME is noteworthy not only as a marker of fermentation progression but also due to its functional implications. While CA has been previously reported to inhibit proliferation through the AKT/GSK-3β/β-catenin signaling pathway in pancreatic cancer cells [35], our data indicate that its methylated derivative, CAME, possesses stronger cytotoxic and pro-apoptotic effects on PANC-1 cells. The structural methylation of CA may improve CAME’s cellular uptake, stability, or target specificity, potentially endowing it with a distinct and more potent mechanism of action [36]. Therefore, the decrease in CA, coupled with the emergence of CAME, may partly explain the enhanced bioactivities of FSLE, providing a mechanistic link between microbial fermentation and the improvement in pharmacological properties.

Further supporting this, Figure 5I shows that CAME inhibited the viability of PANC-1 cells in a concentration-dependent manner. Notably, when the concentration exceeded 100 μg/mL, a significant reduction in cell viability was observed. Specifically, the IC_50_ of CAME was 119.1 μg/mL at 48 h, substantially lower than the 189.6 μg/mL recorded for CA, confirming its greater cytotoxic potency. This result aligns with the hypothesis that methylation improves compound bioactivity, possibly due to enhanced lipophilic interactions and cellular uptake [36].

### 2.6. Effects of CAME on Cell Cycle and Apoptosis in PANC-1 Cells

Cancer typically resists cell death by inhibiting apoptosis and accelerating cell proliferation. Manipulating apoptosis and cell cycle pathways is a crucial strategy in cancer therapy [37]. Compounds capable of overcoming these resistance mechanisms are of considerable interest as potential anticancer agents. To elucidate the effects of CAME on pancreatic cancer cells, we further investigated its impact on the cell cycle and apoptosis in PANC-1 cells using flow cytometry.

**Figure 6:**
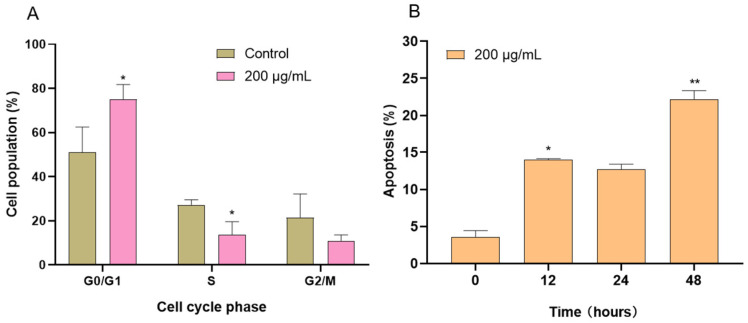
Effects of CAME on the cell cycle and apoptosis of PANC-1 cells. (**A**) CAME arrested PANC-1 cells in the G_0_/G_1_ phase after treatment for 12 h. (**B**) CAME induced apoptosis of PANC-1 cells after treatment for 12, 24, and 48 h. * *p* < 0.05 and ** *p* < 0.01 as compared with control.

Remarkably, at a concentration of 200 μg/mL, CAME demonstrated a significant cell cycle arrest effect, markedly inducing G_0_/G_1_ phase arrest in PANC-1 cells, indicating its inhibitory effect on cell proliferation (Figure 6A). To determine whether this cytostatic effect was accompanied by pro-apoptotic activity, we evaluated apoptosis in PANC-1 cells following CAME treatment. As shown in Figure 6B, at a concentration of 200 μg/mL, the proportion of apoptotic cells increased from 4.4% (0 h) to 13.9% (12 h), 12.2% (24 h), and 21.4% (48 h) with extended treatment time. These results indicate that CAME induces apoptosis, further supporting its potent cytotoxicity against PANC-1 cells.

### 2.7. CAME Pro-Apoptotic Effects by Regulating the Expression of Apoptosis-Related Genes

Apoptosis is a process of programmed cell death that is essential for development and homeostasis in multicellular organisms. Inducing apoptosis is considered an attractive therapeutic approach for cancer treatment due to its lower toxicity to normal tissue [38]. Extensive research has shown that *Bcl-2* family members play a key role in the intrinsic apoptosis pathway by regulating mitochondrial dysfunction [39]. The balance among these family members determines cell fate; thus, modulating the expression of apoptosis-related genes can be used for cancer therapy [40].

**Figure 7:**
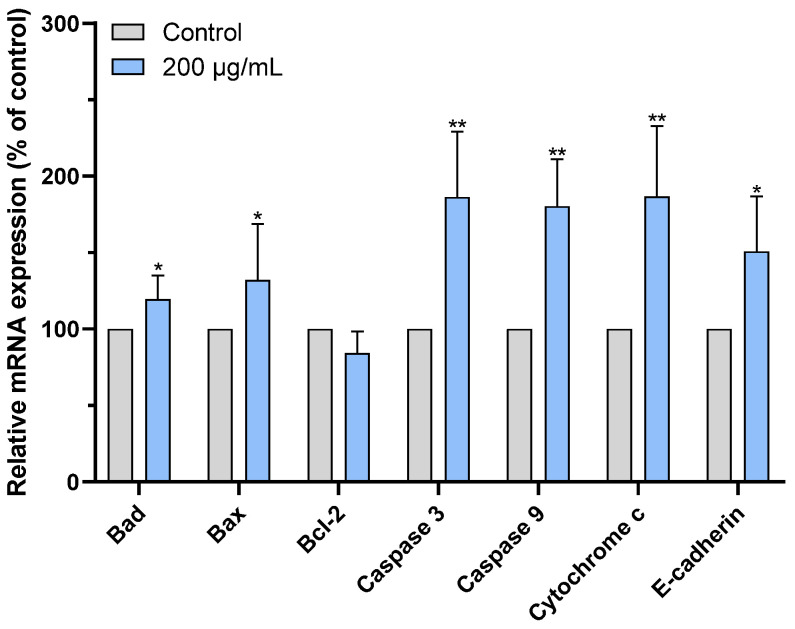
Changes in mRNA levels (the control group set as 100%). CAME induced changes in the mRNA levels of related genes in PANC-1 cells for 5 h at 200 μg/mL. * *p* < 0.05, ** *p* < 0.01 as compared with control.

**Figure 8:**
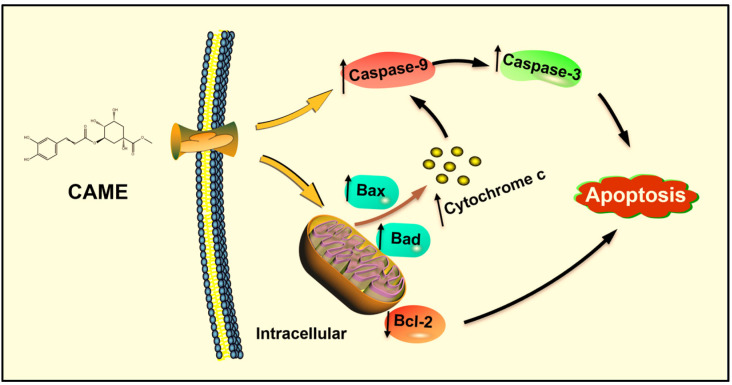
The overall mechanism for the induction of apoptosis in PANC-1 cells by FSLE is summarized in the picture.

To further explore the mechanism underlying the cytotoxic effects of CAME, we examined the expression of apoptosis-related genes in PANC-1 cells. As shown in Figure 7, CAME treatment significantly upregulated the expression of pro-apoptotic genes *Bax* and *Bad*, while downregulating the anti-apoptotic gene *Bcl-2*. The *Bcl-2* gene inhibits apoptosis by suppressing pro-apoptotic proteins such as *Bax*, while *Bad* promotes apoptosis indirectly by binding to *Bcl-2* and preventing its anti-apoptotic effects, thus enhancing *Bax* activity [39]. The increase in the *Bax*/*Bcl-2* ratio indicates *Bax* overexpression, forming *Bax*/*Bax* homodimeric channels that disrupt mitochondrial membrane integrity, promoting the release of *Cytochrome c* from the mitochondria into the cytoplasm, and further activating the caspase cascade, leading to programmed cell death [41]. In addition to modulating the *Bcl-2* family, CAME also upregulated the gene expression of *Cytochrome c*, *Caspase-9*, and *Caspase-3*, the core components of the intrinsic apoptotic pathway. Upon release from mitochondria, *Cytochrome c* binds to apoptotic protease activating factor-1 (APAF-1), forming the apoptosome and activating *Caspase-9*, which in turn cleaves and activates executioner *Caspase-3* [42,43]. These observations indicate that CAME induces apoptosis via the mitochondrial (intrinsic) pathway by orchestrating a cascade of gene expression changes that destabilize mitochondrial integrity and activate caspases (Figure 8).

Notably, our findings are consistent with previous studies on stevia-derived compounds. For example, Khare et al. reported that stevioside increased the *Bax*/*Bcl-2* ratio in triple-negative MDA-MB-231 and SKBR-3 breast cancer cells, promoting apoptosis [12]. Similarly, isosteviol was found to reduce the viability of NUGC-3 gastric cancer cells through mechanisms likely involving mitochondrial dysfunction [13]. These parallels support the notion that stevia glycosides and their derivatives exert anticancer effects, at least in part, through mitochondrial-mediated apoptosis.

In the present study, CAME was detected exclusively in FSLE, suggesting that microbial fermentation may enhance the anticancer activity of stevia by generating bioactive metabolites absent in the non-fermented extract. Our previous work with *L. plantarum* SN13T demonstrated that fermentation could enhance the antioxidant and anti-inflammatory properties of various herbal extracts [44,45,46]. Here, we show that FSLE-derived CAME contributes to selective cytotoxicity against PANC-1 cells by modulating key apoptotic regulators, reinforcing the potential of fermentation biotechnology to amplify the therapeutic value of plant-based compounds.

## 3. Conclusions

This study utilized stevia leaf extract fermented with *L. plantarum* SN13T and determined the optimal fermentation conditions. Under these conditions, FSLE exhibited significant cytotoxicity against PANC-1 cells, outperforming the unfermented extract with minimal toxicity toward HEK-293. FSLE effectively inhibited the clonogenicity, migration, and adhesion of PANC-1 cells. Additionally, it demonstrated superior antioxidant activity in DPPH and ABTS radical scavenging assays, as well as in H_2_O_2_-induced oxidative damage assays, compared to SLE. Furthermore, through HPLC separation followed by structure elucidation using ^1^H-NMR, ^13^C-NMR, and ESI-MS, the active compound was identified as CAME. Subsequent flow cytometry analysis revealed that CAME effectively arrested PANC-1 cells at the G_0_/G_1_ phase and induced apoptosis. At the molecular level, CAME significantly upregulated the expression of *Bax*, *Bad*, *Cytochrome c*, *Caspase-3*, and *Caspase-9* genes in PANC-1 cells, while it downregulated *Bcl-2* gene expression.

In conclusion, FSLE significantly enhances antioxidant and anti-pancreatic cancer cell activity, which is likely related to the production of CAME. CAME may exert its anticancer effects by inducing cell cycle arrest and promoting apoptosis. This study deepened our understanding of the mechanism of action of *L. plantarum* SN13T in herbal extract fermentation and provided a new research perspective on the application of probiotics in natural anti-tumor agents.

## 4. Materials and Methods

### 4.1. Bacterial Cultivation and Fermentation Conditions

*L. plantarum* SN13T was cultured in de Man, Rogosa, and Sharpe (MRS) broth (Merck KGaA, Darmstadt, Germany) under anaerobic conditions at 37 °C for 24 h. After incubation, bacterial cells were harvested by centrifugation and resuspended in an equal volume of sterile phosphate-buffered saline (PBS) solution.

The fermentation method was adapted from previous studies with slight modifications [47]. Briefly, to prepare a 5% (*w*/*v*) SLE, 5 g of dried stevia leaves (Kojima Kampo Co., Ltd., Osaka, Japan) were soaked in 100 mL of distilled water and heated at 105 °C for 30 min. After cooling to room temperature, the extract was centrifuged at 5000× *g* for 15 min, and the resulting supernatant was filtered through a 0.22 μm membrane filter (Advantec Toyo Co., Ltd., Tokyo, Japan).

*L. plantarum* SN13T cells resuspended in PBS were inoculated into the 5% SLE and fermented at 37 °C for 48 h. After fermentation, the supernatant was collected by centrifugation and filtered to obtain sterile FSLE. The soluble solid content was obtained by vacuum drying and used in subsequent experiments, with dosages calculated based on the concentration of soluble solids.

### 4.2. Optimization of Fermentation Conditions for Stevia Leaf Extract

To determine the optimal conditions for producing anticancer substances, the fermentation supernatant of *L. plantarum* SN13T was used as the experimental material; different fermentation times (24, 48, 72 h), temperatures (28, 37 °C), as well as aerobic or anaerobic fermentation, were used. Cells were treated with 250 μg/mL of FSLE and cultured for 48 h. FSLE was solubilized in the RPMI 1640 medium, and the fresh culture medium was used alone as the control. All experiments were performed in triplicate, and average values were calculated.

### 4.3. Cell Culture and Cell Growth Inhibition Assay

The human pancreatic cancer cell line PANC-1 (RBRC, RCB2095) was purchased from the RIKEN BRC Cell Bank, and the non-cancerous human embryonic kidney cell line HEK-293 was kindly provided by Dr. Nakashima Ayumu (Hiroshima University). PANC-1 and HEK-293 cells were cultured in Roswell Park Memorial Institute (RPMI)-1640 and high-glucose Dulbecco’s Modified Eagle’s Medium (DMEM), respectively, at 37 °C with 5% CO_2_. All media were supplemented with 10% fetal bovine serum (FBS, Life Technologies Co., Grand Island, NY, USA) and 1% penicillin/streptomycin (FUJIFILM Wako Pure Chemical Co., Osaka, Japan).

To determine the effects of FSLE, SLE, CAME (Funakoshi Co., Ltd., Tokyo, Japan), and CA (Tokyo Chemical Industry Co., Ltd., Tokyo, Japan) on the proliferation of PANC-1 and HEK-293 cells, an MTT (FUJIFILM Wako Pure Chemical Co., Osaka, Japan) assay was performed. Briefly, cells were seeded in 96-well plates at a density of 1 × 10^5^ cells per well and incubated overnight. The following day, cells were treated with various concentrations of the test samples for a specific period. FSLE was dissolved in RPMI 1640 or DMEM, and fresh culture medium alone served as the control. After treatment, 10 μL of MTT solution (5 mg/mL) was added to each well and incubated for 4 h. The medium was then removed, and 100 μL of solubilization solution (20% SDS + 50% DMF) was added to dissolve the formazan crystals completely. Finally, absorbance was measured at 570 nm using a microplate reader (Varioskan, Thermo Scientific, Vantaa, Finland) [48].

Inhibitory rate (%) = [1 − (A_sample_ − A_blank_)/(A_control_ − A_blank_)] × 100, where A_control_ and A_blank_ are the absorbance values without samples or cells, respectively. Additionally, the half-maximal inhibitory concentration (IC_50_) for PANC-1 cells after 48 h was measured.

### 4.4. Antioxidant Testing

#### 4.4.1. Scavenging DPPH Free Radicals

To assess the effect of SLE and FSLE on DPPH free radicals, a microplate assay was performed following the method described by Retamozo et al. [29]. Briefly, 0.2 mM DPPH solution was prepared in methanol, and equal volumes of the test samples at various concentrations were mixed. The mixtures were shaken and allowed to stand in the dark at room temperature for 30 min. Absorbance was then measured at 517 nm using a microplate reader. In the control group, methanol was used instead of the sample, while the blank group contained methanol without DPPH.

#### 4.4.2. Scavenging ABTS Free Radicals

To better evaluate the antioxidant activity of the samples, their ability to scavenge ABTS free radicals was assessed for a comprehensive evaluation. The assay was performed based on the method previously described by Retamozo et al., with slight modifications [29]. Briefly, a 7.0 mM ABTS solution was mixed with a 2.45 mM potassium persulfate solution at a 1:1 ratio and allowed to react in the dark for at least 12 h. Test samples at various concentrations were then mixed with the ABTS working solution. After shaking, the mixtures were kept in the dark at room temperature for 6 min. Finally, the absorbance was measured at a wavelength of 734 nm.

#### 4.4.3. Antioxidant Activity of HEK-293 Cells

Following the method described by Lopez et al., the antioxidant potential of SLE and FSLE was tested [11]. HEK-293 cells were seeded into a 96-well plate at a density of 1 × 10^5^ cells per well. After 12 h, the cells were incubated with different concentrations of FSLE or SLE for an additional 12 h. Subsequently, the cells were treated with 0.5 mM H_2_O_2_ for 6 h. After incubation, cell viability was assessed using an MTT assay.

### 4.5. Colony Formation Assay

Cells were seeded at a density of 1000 cells per well in a 24-well plate and incubated for 12 h. The cells were then treated with various concentrations of FSLE for an additional 12 h. After treatment, the medium was replaced with a fresh culture medium, and the cells were incubated for approximately 10 days. At the end of the incubation period, the cells were fixed with 0.5 mL of 4% paraformaldehyde solution for 30 min. Subsequently, they were stained with 1% crystal violet solution for 20 min and washed three times with PBS. Finally, images were captured, and the number of colonies was analyzed using ImageJ software (version 1.52, National Institutes of Health, USA). Colonies were defined as cell clusters containing more than 50 cells or occupying an area larger than 1 mm in diameter.

### 4.6. Wound-Healing Assay

PANC-1 cells were seeded in a 6-well plate and incubated overnight. A vertical scratch was made on the bottom of the well using a 200 μL pipette tip, and the wells were washed with PBS to remove the detached cells. Subsequently, a medium containing various concentrations of FSLE was added. FSLE was directly dissolved in the RPMI 1640 medium, and cells treated with fresh culture medium alone were used as the control group. At 0, 12, 24, and 48 h post-treatment, images were captured using a microscope (Olympus IX71, Tokyo, Japan).

### 4.7. Migration Assay

A total of 100 μL of cell suspension containing 1% FBS and various concentrations of FSLE, the mixture was added to the upper chambers of transwell inserts (8 μm pore size). The lower chambers were filled with fresh medium containing 20% FBS. After 24 h of incubation, the cells were fixed with 4% paraformaldehyde for 30 min and stained with 1% crystal violet for 10 min. The migration chambers were then washed three times with PBS, and non-migrated cells on the upper side of the membrane were removed with a cotton swab. Finally, images were captured under a microscope, and the number of cells was quantified using ImageJ software.

### 4.8. Extraction and Identification of Active Compounds Produced in FSLE

To compare the components of SLE and FSLE, a pre-HPLC analysis was conducted based on a previous study [46]. Briefly, both SLE and FSLE were mixed with 1/5 volume of 5 M sodium chloride solution and extracted with three volumes of a chloroform–methanol mixture. The methanol layer was collected and subjected to silica gel column chromatography, using hexane, ether, ethyl acetate, and methanol for sequential elution. Bioactivity was verified by MTT assay and further analyzed by HPLC.

HPLC analysis was performed using a JASCO system (JASCO Corporation, Tokyo, Japan) equipped with a YMC-Pack ODS-AQ column (5 µm, 30 nm, Φ = 3 mm, L = 150 mm; YMC, Kyoto, Japan). The mobile phase consisted of water with 0.1% trifluoroacetic acid (solvent A) and acetonitrile (solvent B). The elution program was as follows: 0–5 min, 5% B; 5–15 min, 5–30% B; 15–25 min, 30–40% B. The flow rate was set at 1 mL/min, and the column temperature was maintained at room temperature. A 5.0 μL aliquot of each sample was injected, and elution was monitored at 270 nm. The chromatograms of SLE and FSLE were compared to identify peaks corresponding to new compounds produced during fermentation. The purified compound was identified using a combination of ESI–MS as well as ^1^H-NMR and ^13^C-NMR spectra. The MS spectra were captured on a Thermo Fisher Scientific LTQ Orbitrap XL. The parameters for EI mode were an ion-source temperature of 250 °C, an electron energy of 70 eV, and a filament current of 300 µA. ^1^H-NMR and ^13^C-NMR spectra were captured on a JEOL JNM-LA500 spectrometer (JEOL Ltd., Tokyo, Japan) at 500 MHz. Finally, the identified compound was confirmed by HPLC using the analytical standard, and its concentrations were determined by external standard curves based on absorbance at 270 nm.

### 4.9. Flow Cytometry Analysis

#### 4.9.1. Cell Cycle Analysis

To assess the cell cycle distribution, the Cell Cycle Assay Solution Blue (C549, Dojindo, Tokyo, Japan) was used in accordance with the manufacturer’s instructions. Briefly, PANC-1 cells in good growth condition were seeded at 6 × 10^5^ cells per well in a 6-well plate and incubated overnight. The cells were then treated with CAME for 12 h. After treatment, cells were digested with trypsin, washed with PBS, and collected by centrifugation. They were fixed in 70% ethanol at 4 °C overnight. After centrifugation and PBS washing, 5 μL of the Cell Cycle Assay Solution was added and incubated at 37 °C for 15 min. Finally, the DNA content was measured using flow cytometry (FACS Canto II, Becton Dickinson, Franklin Lakes, NJ, USA) based on staining intensity to determine the distribution of cell cycle phases.

#### 4.9.2. Cell Apoptosis Analysis

Apoptosis was evaluated using the Annexin V-FITC Apoptosis Detection Kit (15342-54 Nacalai Tesque, Kyoto, Japan) by measuring Annexin V- and propidium iodide (PI)-positive cells, according to the manufacturer’s instructions. Briefly, PANC-1 cells in good growth condition were seeded at 6 × 10^5^ cells per well in a 6-well plate and incubated overnight. The cells were then treated with CAME for 12, 24, and 48 h. After treatment, cells were detached using trypsin without EDTA, collected by centrifugation, and washed three times with pre-chilled PBS. Subsequently, 5 μL of Annexin V/Alexa Fluor 488 and PI staining solution were added to the cells and incubated in the dark at room temperature for 15 min. Flow cytometric analysis was completed within 1 h.

### 4.10. RNA Extraction and qRT-PCR Analysis

PANC-1 cells were seeded at 6 × 10^5^ cells per well in a 6-well plate and incubated overnight. The cells were then treated with CAME for 5 h before being collected. Total RNA was isolated from the cells using the NucleoSpin RNA Plus kit (Macherey-Nagel GmbH & Co. KG, Duren, Germany). Reverse transcription was performed in accordance with the manufacturer’s instructions using the ReverTra Ace qPCR RT Master Mix with gDNA Remover (Toyobo, Japan). qRT-PCR was conducted using the KAPA SYBR Fast qPCR Kit (Kapa Biosystems, Woburn, MA, USA) on a PikoReal Real-Time PCR System (Thermo Fisher Scientific, Inc., Waltham, MA, USA).

The qPCR conditions were as follows: an initial 2 min at 95 °C, followed by 40 cycles of 5 s at 95 °C and 10 s at 60 °C. Relative transcription levels were normalized to the housekeeping gene (*GAPDH*). The ΔΔCT method was used to analyze the gene expression of *Bax*, *Bad*, *Bcl-2*, *Caspase-3*, *Caspase-9*, and *E-cadherin*. The primer sequences used in this experiment are listed in Table 1.

### 4.11. Statistical Analysis

All experiments were conducted with three independent biological replicates. Data were expressed as means ± standard deviation (SD). Statistical significance between the two groups was assessed using an independent samples *t*-test. Statistical differences among multiple groups were evaluated using a one-way analysis of variance (ANOVA), followed by a post hoc Tukey test. A *p*-value < 0.05 was considered statistically significant. Analyses were implemented in SPSS 22.0 (IBM Corp., Armonk, NY, USA) and GraphPad Prism 9.0 (San Diego, CA, USA).

## Figures and Tables

**Table 1:** Primers used in qPCR experiments.

Genes	5′-3′ Primer Pairs	Primer Sequences (5′-3′)
*E-cadherin*	Forward	GCCTCCTGAAAAGAGAGTGGAAG
	Reverse	TGGCAGTGTCTCTCCAAATCCG
*Bcl-2*	Forward	ATCGCCCTGTGGATGACTGAGT
	Reverse	GCCAGGAGAAATCAAACAGAGGC
*Bax*	Forward	TCAGGATGCGTCCACCAAGAAG
	Reverse	TGTGTCCACGGCGGCAATCATC
*Bad*	Forward	CCAACCTCTGGGCAGCACAGC
	Reverse	TTTGCCGCATCTGCGTTGCTGT
*Caspase-3*	Forward	GGAAGCGAATCAATGGACTCTGG
	Reverse	GCATCGACATCTGTACCAGACC
*Caspase-9*	Forward	GTTTGAGGACCTTCGACCAGCT
	Reverse	CAACGTACCAGGAGCCACTCTT
*GAPDH*	Forward	GTCTCCTCTGACTTCAACAGCG
	Reverse	ACCACCCTGTTGCTGTAGCCAA
*Cytochrome* *c*	Forward	AAGGGAGGCAAGCACAAGACTG
	Reverse	CTCCATCAGTGTATCCTCTCCC

## Data Availability

All data are reported in the manuscript/Supplementary Material.

## Supplementary Materials

The following supporting information can be downloaded at: https://www.mdpi.com/article/10.3390/ijms26094186/s1.
