# Supplementary material for: Stevia Leaf Extract Fermented with Plant-Derived Lactobacillus plantarum SN13T Displays Anticancer Activity to Pancreatic Cancer PANC-1 Cell Line
Source: Int J Mol Sci. 2025 Apr 28;26(9):4186. doi: 10.3390/ijms26094186 (PMC12071683; doi:10.3390/ijms26094186)
Supplement: Supplementary file 1 [file ijms-26-04186-s001.zip › ijms-3579839-supplementary.pdf]

S1 (a)

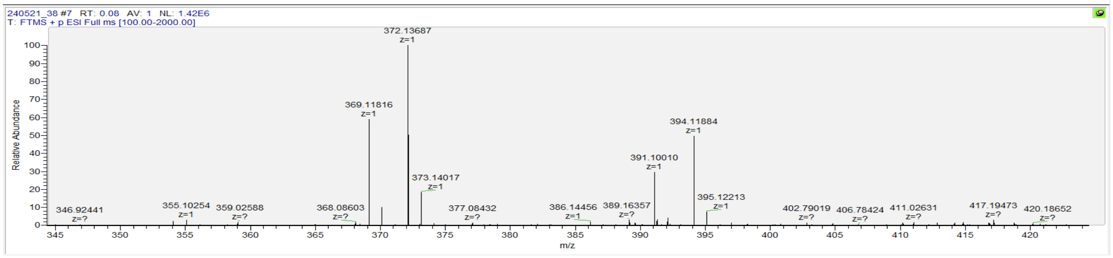

S1 (b)

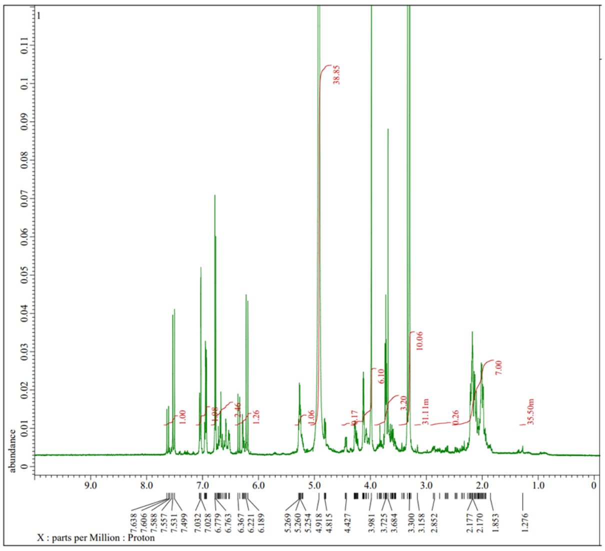

S1 (c)

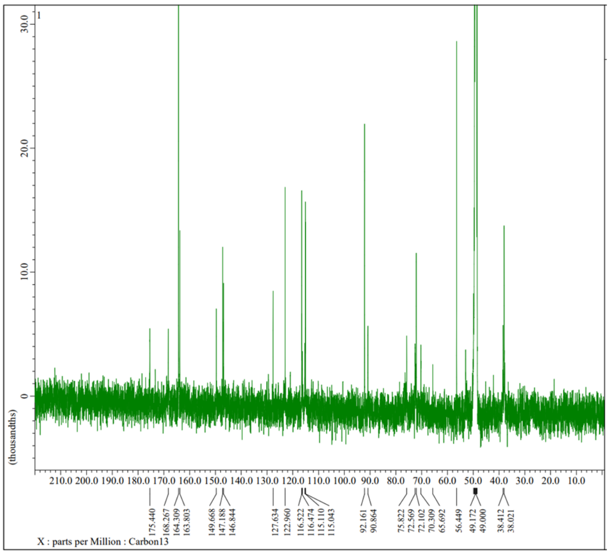

Figure S1: (a) ESI-MS spectra, (b) ^{1}H-NMR spectra, and (c) ^{13}C-NMR spectra of chlorogenic acid methyl ester, purified from FSLE.
